# The Role of Burnout Dimensions, Job Stress, and Work–Life Balance in Multisite Musculoskeletal Pain Among Academicians in Türkiye: A Cross-Sectional Study

**DOI:** 10.3390/healthcare14111475

**Published:** 2026-05-27

**Authors:** Erdi Kayabınar, Zeynep İclal Sağ, Büşra Kayabınar, Ebrar Atak

**Affiliations:** Department of Physiotherapy and Rehabilitation, Faculty of Health Sciences, University of Yalova, Yalova 77200, Türkiye; zeynep.sag@yalova.edu.tr (Z.İ.S.); busra.kayabinar@yalova.edu.tr (B.K.); ebrar.atak@yalova.edu.tr (E.A.)

**Keywords:** musculoskeletal disorders, job stress, burnout, work–life balance, academicians

## Abstract

**Background**: Burnout is common among academicians and is linked to both mental and physical health problems. However, its connection to musculoskeletal disorders (MSDs) is not well understood. This study aimed to investigate how burnout, job stress, and work–life balance are related to multisite musculoskeletal pain in academicians. **Methods**: In this cross-sectional study, 99 academicians at Yalova University completed validated instruments assessing MSDs, burnout, work-related stress, and work–life balance. The study employed a voluntary convenience sampling method and included academicians who agreed to participate. Data were analysed using correlation and ordinal regression analysis. **Results**: The one-year prevalence of MSDs was 88.9% and most commonly involved the neck, lower back, and upper back. While overall burnout scores were not significantly related to the number of painful regions, the number of painful regions was positively correlated with job stress and computer usage and negatively with work–life balance and personal accomplishment (*p* < 0.05). Ordinal regression analysis showed that job stress, computer usage, and impaired work–life balance were associated with greater multisite pain burden (*p* < 0.01). **Conclusions**: Although overall burnout was not associated with multisite musculoskeletal pain, psychosocial factors such as job stress and work–life balance were significantly related to multisite pain. These findings suggest that occupational stress may be linked to physical health outcomes before the full manifestation of burnout develops. Addressing job stress and work–life balance may help reduce MSDs among academicians.

## 1. Introduction

Burnout has increasingly been recognized as a major occupational health problem, particularly in professions characterized by high interpersonal demands and complex role expectations, such as academia [1]. According to Maslach and colleagues, burnout is a psychological syndrome consisting of emotional exhaustion, depersonalization, and reduced personal accomplishment that develops as a response to chronic occupational stress [2]. Academicians face unique challenges because their work involves teaching, research, administration, supervising students, and engaging with society. Balancing these responsibilities, along with high expectations and limited time, often leads to significant stress and burnout [3,4].

Stress, burnout, and work–life balance are closely related yet conceptually distinct constructs in the field of occupational health research. Stress is a psychological and physiological reaction in response to perceived job demands that are greater than the individual’s coping resources; it can be acute or temporary. Burnout, on the other hand, is known as a chronic psychological syndrome that occurs in response to extended interpersonal stressors at work [5,6]. In contrast, work–life balance is an individual experience, representing the extent to which a person can create harmony between competing demands related to their work and lives, and is considered a wider contextual and changeable factor that has an impact on well-being [7]. Burnout is a negative outcome of chronic stress exposure; conversely, work–life balance may be a protective factor reducing the impact of work-related stress leading to burnout [8].

Burnout is common among academicians and is linked to several organizational and psychosocial factors. These include heavy teaching loads, lack of support from institutions, limited research resources, poor reward systems, staff shortages, and difficult work environments [9,10]. Academic work often means long hours, tight deadlines, extra paperwork, and frequent interruptions. These demands can increase emotional exhaustion and stress. As a result, burnout can harm mental health, job satisfaction, productivity, and the overall quality of professional life [11,12]. Burnout is a significant concern for academicians, with an estimated prevalence of 10.7% in a university-based study [13]. In addition, some studies have reported prevalence rates ranging from 9% to 23.8% in academic populations [14], and many academicians continuously had high scores on burnout dimensions such as emotional exhaustion and depersonalization [15].

Recent research has shown that burnout affects not only mental health but also has a strong connection to physical health problems [16]. Systematic reviews and longitudinal studies provide evidence that work-related psychosocial factors are independently associated with the development and progression of musculoskeletal disorders [16,17]. Individuals experiencing burnout frequently report fatigue, sleep disturbances, somatic complaints, and impaired recovery capacity. From a physiological perspective, chronic occupational stress and burnout may trigger sustained activation of stress-response systems, including dysregulation of the hypothalamic–pituitary–adrenal axis and increased sympathetic nervous system activity [18]. These mechanisms may lead to prolonged muscle tension, altered neuromuscular activation patterns, and increased biomechanical loading, thereby contributing to musculoskeletal complaints [19].

Musculoskeletal disorders (MSDs) are among the most common occupational health problems worldwide and are characterized by pain or discomfort affecting muscles, joints, tendons, ligaments, and spinal structures. Although MSDs are traditionally associated with biomechanical and ergonomic risk factors such as repetitive movements and poor posture, recent research has highlighted the role of psychosocial determinants in their development [19,20]. In academic settings, prolonged sitting, standing, and awkward postures coexist with psychosocial stressors, creating a multifactorial risk environment for MSDs [21,22].

The interaction between burnout and MSDs is increasingly discussed but remains insufficiently understood. Burnout-related emotional exhaustion, which is considered the core component of the syndrome, has been identified as a sensitive indicator of chronic work stress and may play a critical role in the development of physical symptoms [5]. The reasons for the association between psychosocial factors and MSDs include the triggering of physiological responses by psychosocial stressors, which subsequently lead to biochemical stress responses such as muscle tension, co-activation, and increased loading on the musculoskeletal system [23,24,25]. Furthermore, work–life imbalance and academic stress, which are strongly associated with burnout, may intensify physiological stress responses and exacerbate musculoskeletal discomfort. Academicians often struggle to maintain work–life balance due to the simultaneous management of multiple professional and personal roles, and this imbalance has been linked to both psychological strain and physical health problems [26,27].

Despite increased recognition of the psychosocial aspects of MSDs, most research on academicians continues to focus primarily on physical and ergonomic risk factors. The combined effects of burnout, academic stress, and work–life balance have received limited attention. Furthermore, given the multidimensional nature of burnout, the differential impact of its components on musculoskeletal health among academicians remains insufficiently explored. Therefore, this study aimed to investigate the relationship between burnout and musculoskeletal disorders while also examining the roles of job stress and work–life balance among academicians.

## 2. Materials and Methods

### 2.1. Study Design and Participants

This cross-sectional study was conducted among academicians working at Yalova University between January 2025 and July 2025, following approval from the Yalova University Non-Interventional Clinical Research Ethics Committee, dated 4 December 2024, with number 2024/292, and institutional permission from Yalova University. The study protocol was also registered at ClinicalTrials.gov (ID: NCT07276516). The study was registered to ensure transparency and improve the accessibility of the study details. All volunteers who participated in the study were informed about the study and provided their written informed consent. The study was performed in accordance with the ethical standards of the 1964 Declaration of Helsinki and its later amendments or comparable ethical standards.

The study population consisted of all academicians working at Yalova University. The study employed a voluntary convenience sampling method, in which academicians who met the inclusion criteria and agreed to participate were recruited. As participation was based on voluntary recruitment from a single institution, no a priori sample size calculation was performed.

The inclusion criteria for the study were volunteering to participate, having worked as academic staff at Yalova University for the past six months, and being able to understand and speak Turkish. Individuals who wished to withdraw from the study, those with a history of fracture or soft tissue injury in any body region within the past 12 months, those with congenital spinal disorders, scoliosis, rheumatoid diseases, cancer, surgery, chronic internal organ pain, long-term use of analgesics or psychiatric medications, and those working in departments without students were excluded from the study.

The demographic information of the academicians who volunteered to participate in the study was first recorded using a question-and-answer method. The demographic data included age, sex, height, weight, academic title, weekly working hours, and weekly computer usage. After the demographic information was recorded, the participants completed the questionnaires listed below, and the results were recorded.

### 2.2. Outcome Measures

In this study, two main study variables were considered: multisite musculoskeletal pain (number of painful body regions) and burnout dimensions. Job stress and work–life balance were treated as explanatory variables. The following instruments were used to assess these outcomes:

Burnout was assessed using the Maslach Burnout Inventory (MBI), developed by Maslach and Jackson in 1981 [2]. The inventory has been culturally adapted and validated for use among academicians in Türkiye [28,29]. The MBI consists of 22 items and evaluates burnout across three dimensions: emotional exhaustion, depersonalization, and personal accomplishment. The emotional exhaustion subscale includes nine items assessing fatigue, emotional depletion, and reduced energy; the depersonalization subscale contains five items reflecting detached or impersonal attitudes toward recipients of professional services; and the personal accomplishment subscale comprises eight items measuring perceived competence and achievement. Items are rated on a 7-point Likert scale ranging from 0 to 6. Separate scores were calculated for each subscale. Higher scores in emotional exhaustion and depersonalization, or lower scores in personal accomplishment, indicate higher levels of burnout [29].

Musculoskeletal disorders were assessed using the Extended Nordic Musculoskeletal Questionnaire (NMQ-E). The Standardized Nordic Musculoskeletal Questionnaire was originally introduced by Kuorinka and colleagues in 1987 as a screening instrument for musculoskeletal disorders and was later expanded by Dawson and colleagues to provide more detailed information regarding prevalence and functional impact [30,31]. The NMQ-E includes questions addressing symptoms across nine body regions: neck, upper back, shoulders, elbows, hands/arms, lower back, hips, knees, and feet/ankles. A body diagram was provided to facilitate accurate reporting of pain or discomfort locations, and responses were recorded as dichotomous (yes/no). The Turkish version of the questionnaire has demonstrated acceptable validity and reliability [32]. In this study, multisite musculoskeletal pain was defined as the total number of body regions in which participants reported pain or discomfort.

Work-related stress was evaluated using the University Academic Staff Work Stress Scale (UASWSS), developed by Balcı in 1992 and validated for reliability and validity. The scale consists of 24 items with total scores ranging from 0 to 120. Higher scores reflect lower job satisfaction and greater perceived work-related stress [33].

Work–life balance was assessed using the Work–Life Balance Scale (WLB), developed by Taşdelen-Karçkay and Bakalım. The scale contains 8 items rated on a 7-point Likert scale ranging from “strongly disagree” to “strongly agree.” Lower scores indicate disruption in work–life balance [34].

Cronbach’s alpha coefficients for the scales were not calculated in the current study, as item-level data were not recorded. However, the reliability of the instruments has been previously established in validation studies, with reported Cronbach’s alpha values indicating acceptable to high internal consistency. A summary of the measurement instruments used in this study is presented in Table 1.

### 2.3. Statistical Analysis

The data were analysed using SPSS version 26.0 (SPSS Inc., Chicago, IL, USA). Categorical variables are presented as frequencies and percentages, while continuous variables are expressed as means and standard deviations. Group comparisons were performed using the Pearson chi-squared test or Fisher’s exact test for categorical variables and the independent samples *t*-test for continuous variables. Pearson correlation analysis was conducted to examine the relationships between variables.

Multisite musculoskeletal pain was defined as the number of body regions in which participants reported pain and was categorized into five ordered groups (0, 1, 2, 3, and ≥4 regions). Due to the ordinal nature of this outcome variable, ordinal logistic regression analysis (proportional odds model) was performed to identify factors associated with increasing pain burden. The ordinal outcome variable was treated as a cumulative logit model with increasing categories representing greater pain burden. Variables included in the regression model were selected based on theoretical relevance and the results of correlation analysis. The proportional odds assumption was evaluated using the test of parallel lines. Multicollinearity among independent variables was assessed using the variance inflation factor (VIF) and tolerance values, and no evidence of multicollinearity was observed. Additionally, one-way ANOVA was used to compare test scores across pain categories, and the Tamhane T2 test was applied for post hoc comparisons. A *p*-value of less than 0.05 was considered statistically significant.

### 2.4. Reporting Guideline

This study is reported in accordance with the Strengthening the Reporting of Observational Studies in Epidemiology (STROBE) statement, and the STROBE checklist is provided as Supplementary Material [35]. All relevant STROBE checklist items were addressed throughout the manuscript.

## 3. Results

A total of 620 academicians working at Yalova University were assessed for eligibility. Of these, 485 were excluded based on the predefined inclusion and exclusion criteria, resulting in 135 eligible participants. The relatively high exclusion rate is primarily attributable to the strict inclusion and exclusion criteria, including the presence of health conditions, not actively working in departments with students, or having less than six months of academic experience. Among the eligible academicians, 36 declined to participate after reviewing the questionnaire’s content. Consequently, 99 academicians were included in the final analysis.

No data were lost during the study. All the participants completed the assessments, and the dataset was fully analysed. Consequently, the study was completed with 99 academicians participating. Among the academicians who participated in the study, 41 (41.4%) were women with a mean age of 37 ± 7 years (min: 27; max: 59), and 58 (58.6%) were men with a mean age of 41 ± 8 years (min: 28; max: 60). The distribution of participants across academic specialties was heterogeneous: 36.4% were affiliated with vocational schools, 27.3% with social sciences (law, economics and administrative sciences, and humanities), 24.3% with health-related fields (health and sports sciences), and 12.1% with technical sciences (engineering). Additional demographic characteristics of the participants are presented in Table 2. When test results were compared between male and female academicians, only job stress was significantly higher among female academicians (*p* < 0.05). Moreover, the impacts of burnout and work–life balance were similar between the groups (*p* > 0.05) (Table 2). Additionally, MSD findings are summarized in Table 2. In both female and male academicians, the highest pain prevalence was observed in the neck, upper back, and lower back regions. A gender-based comparison showed a statistically significant difference only in the prevalence of shoulder pain (*p* < 0.05).

Correlation analyses showed that the number of painful body regions was positively associated with job stress and computer usage duration and negatively associated with work–life balance (*p* < 0.05). This finding suggests that occupational strain is associated with more widespread musculoskeletal symptoms. Additionally, a weak negative association was observed between the number of painful regions and the personal accomplishment subscale of burnout. No significant associations were found with other burnout dimensions (*p* > 0.05). (Table 3).

Table 4 presents the intercorrelations among the main study variables. Work-related stress was positively correlated with emotional exhaustion (r = 0.339, *p* = 0.001) and depersonalization (r = 0.241, *p* = 0.016) and negatively correlated with work–life balance (r = −0.412, *p* < 0.001). Emotional exhaustion was positively correlated with depersonalization (r = 0.413, *p* < 0.001) and negatively correlated with personal accomplishment (r = −0.332, *p* < 0.001) and work–life balance (r = −0.229, *p* = 0.023). No significant correlations were observed between depersonalization and the other variables (*p* > 0.05). These findings indicate that burnout dimensions are related to other psychosocial variables, although they were not directly associated with multisite pain.

Ordinal logistic regression analysis demonstrated that job stress, work–life balance, and computer usage duration were significantly associated with the number of painful body regions (*p* < 0.05) (Table 5). Higher job stress and longer computer usage duration were associated with an increased likelihood of reporting a greater number of painful regions, whereas better work–life balance was associated with a lower likelihood of multisite pain. In contrast, burnout dimensions, including emotional exhaustion, depersonalization, and personal accomplishment, were not significantly associated with the number of painful regions in the adjusted model (*p* > 0.05). The overall model demonstrated a good fit (χ^2^ = 71.818, *p* < 0.001) and explained a substantial proportion of variance (Nagelkerke R^2^ = 0.558). The proportional odds assumption was satisfied (Test of Parallel Lines, *p* = 0.342).

Furthermore, comparisons based on the number of painful regions indicated that academicians with a higher number of painful regions had significantly more impaired work–life balance and job stress (*p* < 0.05) (Table 6).

## 4. Discussion

This study aimed to investigate the relationship between burnout, job stress, work–life balance, and multisite musculoskeletal pain among academicians. One of the most important findings of this study is that although overall burnout scores were not significantly associated with multisite musculoskeletal pain in the regression analysis, job stress, impaired work–life balance, and computer usage duration were significantly associated with the number of painful body regions. However, correlation analyses showed that these variables are related to each other and might influence one another. Notably, job strain and impaired work–life balance were most strongly associated with the number of painful body regions, suggesting a potential role in the physical symptom burden of academicians. These findings suggest that musculoskeletal complaints in academic settings may be more closely related to broader psychosocial stressors than to burnout as a composite construct. Previous studies conceptualize burnout as a progressive, multidimensional process in which emotional exhaustion precedes depersonalization and reduces personal accomplishment [36]. Therefore, the absence of a relationship between overall burnout scores and multisite pain in the present study does not imply the absence of burnout-related effects but rather suggests early-stage manifestations of burnout.

Burnout is a condition frequently observed in service-oriented professions and is characterized by persistent fatigue, emotional depletion, loss of motivation, and reduced professional effectiveness [10]. Maslach and Jackson described burnout as a syndrome involving physical, emotional, and cognitive exhaustion, accompanied by feelings of helplessness, negative self-perception, and adverse attitudes toward work and others [2,37]. Emotional exhaustion is considered the central component of burnout, encompassing experiences such as emotional depletion, hopelessness, passivity, and diminished enthusiasm. Depersonalization refers to emotional distancing from service recipients, while reduced personal accomplishment pertains to negative self-evaluations and perceived professional inefficacy [38]. Given the continuous interpersonal interaction and multifaceted role demands inherent in academia, burnout is a prevalent phenomenon in this professional group and has been linked to decreased productivity, reduced educational quality, and broader societal implications [10,11]. However, in the regression analysis of the current study, burnout and its subdimensions were not significantly associated with multisite musculoskeletal pain. This may indicate that while burnout is an important occupational construct, it may not be the most directly associated psychosocial factor when other related variables such as job stress and work–life balance are considered simultaneously.

The present findings support the conceptualization of work-related stress as a psychosomatic phenomenon. The significant associations among work-related stress, impaired work–life balance, and musculoskeletal complaints may be explained through physiological stress mechanisms. Chronic occupational stress and burnout have been associated with dysregulation of the hypothalamic–pituitary–adrenal axis, increased cortisol levels, and sustained muscle tension [18]. These physiological alterations may lead to changes in neuromuscular activation patterns, increased biomechanical loading, and impaired recovery processes, thereby contributing to the development and persistence of musculoskeletal pain. As a result, these mechanisms may be associated with the presence or severity of musculoskeletal pain [16]. In addition, correlation analyses showed strong intercorrelations between job stress and emotional exhaustion as well as work–life balance, supporting the notion that these psychosocial factors may influence each other in a wider sense. Although the regression analysis showed non-significant effects for emotional exhaustion, its associations with other psychosocial variables suggest that it may act as an indirect factor within a broader psychosocial framework linking occupational stress and musculoskeletal disorders.

Another key finding of this study is the strong relationship between work–life balance and multisite musculoskeletal pain. Work–life imbalance may not directly represent full burnout but can contribute to emotional exhaustion, which is considered an early stage and a key factor of burnout. This finding supports the view that burnout can be understood as a continuum rather than a strictly dichotomous condition. In this context, impaired work–life balance serves as an early warning signal of occupational strain preceding more severe burnout dimensions, such as depersonalization and reduced personal accomplishment [7,8].

The different aspects of burnout might help explain why overall burnout scores are not linked to pain in multiple body areas. Emotional exhaustion means feeling drained, depersonalization is emotional distance, and reduced personal accomplishment is about how people judge their own work [39]. These aspects may not appear at the same time or be affected by work stress in the same way. In academic environments, people might stay engaged and committed to their work even when they feel emotionally exhausted. This could explain why depersonalization and reduced personal accomplishment were not strongly associated with musculoskeletal problems in this study.

Musculoskeletal disorders are recognized as multifactorial conditions arising from the interplay of biomechanical, individual, and psychosocial factors. Although the earlier studies conducted on academicians focused on the role of physical risk factors, such as ergonomic conditions and workload, recent studies have emphasized the importance of psychosocial factors [20]. This study extends previous findings by suggesting that burnout-related psychosocial factors, including job stress and impaired work–life balance, may represent key pathways through which occupational stress is related to the physical symptom experience of academicians [19,40].

Earlier studies also found a high rate of musculoskeletal complaints, and this study showed that the neck, lower back, and upper back were the most affected areas. These results indicate that these symptoms should not be viewed only from a biomechanical perspective. The links between job stress, work–life imbalance, and pain in multiple areas support the dual-pathway model, which suggests that both physical demands and psychosocial stressors affect musculoskeletal health [41,42]. Regression analysis showed that job stress, work–life balance problems, and long hours of computer use all played a significant role in where pain occurred, highlighting the importance of work-related psychosocial factors.

Academic environments are increasingly characterized by heightened performance pressure, complex roles, and evolving institutional expectations. These factors collectively contribute to elevated occupational stress and a greater risk of burnout [10,43]. Specific contributors, including publication pressure, administrative workload, limited institutional support, and role conflict, have been shown to intensify job-related stress among academicians. The present findings are consistent with existing literature, demonstrating high levels of occupational stress and a strong association between stress and multisite musculoskeletal pain [44]. Notably, female academicians reported higher levels of job stress, which may partially account for the observed gender differences in shoulder pain prevalence.

These findings highlight the importance of addressing work-related psychosocial factors, particularly job stress and work–life balance, in occupational health strategies targeting academicians. Strategies such as optimizing workload, clarifying role expectations, enhancing institutional support, and promoting work–life balance may contribute to improvements in both mental and physical health [45]. This mirrors the results of a recent systematic review from the pharmacy sector, which determined that burnout, stress, and workload were significant correlates of job satisfaction and retention, comprising ~24% of reported factors explaining pharmacist turnover behaviour, thus further reinforcing psychosocial stressors as having an overlapping impact on professionals in high-demand work environments [46].

In addition to intended organizational interventions, integrative mind–body protocols can provide additional benefits in preventing or minimizing work-related musculoskeletal impairment. Yoga-based interventions have previously been found to be effective in reducing anxiety and improving stress-related outcomes, which are associated with musculoskeletal symptoms [47]. Structured asana protocols for managing neck, shoulder, and wrist pain have been suggested, specifically targeting professionals engaged in occupations requiring heavy physical work from the upper body [48]. Although those integrative approaches have been mostly studied among dentists, a similar approach may be applicable in academia to psychosocial stress and/or musculoskeletal ailments. Further research is warranted to investigate the practicality and viability of yoga-based protocols in our academic population.

Although several associations reached statistical significance, their clinical relevance should be interpreted with caution. For example, while job stress and computer use were significantly associated with multisite pain, the magnitude of these effects should be weighed against their practical implications. In contrast, work–life balance and job stress demonstrated relatively larger effect sizes, suggesting greater clinical relevance compared to burnout subdimensions. In summary, this study identifies job stress and impaired work–life balance as the key psychosocial factors associated with musculoskeletal complaints among academicians. Although pain burden was not directly associated with burnout dimensions in the regression analysis, the intercorrelations in the current study suggest that emotional exhaustion may still be indirectly impactful via its relationships with job stress and work–life imbalance. These findings support the view that occupational strain is a multidimensional construct involving both psychological and physical components.

## 5. Limitations

This study has several limitations that should be considered when interpreting the findings. Some academicians may have declined to participate due to concerns about the potential impact of their responses on their professional standing, which may have introduced selection bias and led to an underestimation of burnout-related symptoms. As the study was based on self-reported data and employed a cross-sectional design, causal relationships between psychosocial factors and musculoskeletal complaints cannot be established. Moreover, the cross-sectional design precludes establishing the direction of causality; it is possible that musculoskeletal pain contributes to increased stress, impaired work–life balance, and burnout-related symptoms, indicating potential reverse causation. Job stress and impaired work–life balance were prominent in this sample, which may indicate that participants were more likely to experience early manifestations of burnout rather than the full syndrome. Additionally, as the study was conducted at a single centre and relied on self-reported questionnaires to assess musculoskeletal symptoms, the generalizability of the findings may be limited, and the use of self-reported measures may introduce common method bias. Moreover, the absence of objective clinical assessment of musculoskeletal disorders may limit the accuracy of self-reported symptoms and the ability to confirm the presence of clinically diagnosed conditions. No formal a priori sample size calculation was performed, which may limit the statistical power of the study. Furthermore, the relatively small sample size, together with the number of predictors included in the regression model, may have increased the risk of overfitting and limited the ability to detect small effect sizes. Not all potential confounding variables were included in the regression model, which may have influenced the observed associations. Factors such as age, academic specialty, and workload characteristics may have influenced both psychosocial variables and musculoskeletal outcomes. However, given the relatively small sample size and the number of predictors already included, adding further variables could have increased the risk of overfitting and unstable estimates. In addition, sparse data across some categories may have further limited model stability. Therefore, these variables were not included, and residual confounding cannot be excluded. Future studies with larger sample sizes should consider including these variables in multivariable models.

## 6. Conclusions

This study highlights work-related stress and impaired work–life balance as key psychosocial factors associated with multisite musculoskeletal pain among academicians. Although overall burnout scores were not linked to pain in multiple areas, the strong associations with job stress and work–life balance suggest that early signs of burnout may be associated with physical health outcomes through stress-related mechanisms, although causal relationships cannot be established due to the cross-sectional design. These results may support the idea that both physical and psychological factors, such as job stress and work–life balance, play a role in musculoskeletal symptoms in academic settings. Spotting and managing burnout early, by improving workload, support, and work–life balance, may help lower both mental distress and physical symptoms. Future studies should focus on longitudinal designs to better understand the temporal relationships between psychosocial factors and musculoskeletal symptoms, as well as intervention studies targeting burnout-related factors.

## Figures and Tables

**Table 1:** Measurement instruments used in the study.

Instrument	Abbreviation	No. of Items	Measured Domain	Cronbach’s α	Turkish Validation Study
Extended Nordic Musculoskeletal Questionnaire	NMQ-E	9 regions	Presence of musculoskeletal symptoms (12-month prevalence)	0.78	Alaca et al., 2019 [32]
University Academic Staff Work Stress Scale	UASWSS	24	Perceived occupational stress and job satisfaction	0.85	Balcı, 1992 [33]
Maslach Burnout Inventory	MBI	22	Burnout dimensions: emotional exhaustion (EE), depersonalization (DEP), personal accomplishment (PA)	0.88 (EE) 0.78 (DEP) 0.74 (PA)	İnce & Şahin, 2015 [29]
Work–Life Balance Scale	WLB	8	Perceived balance between work and personal life	0.92	Taşdelen-Karçkay & Bakalım, 2017 [34]

Abbreviations: NMQ-E, Extended Nordic Musculoskeletal Questionnaire; UASWSS, University Academic Staff Work Stress Scale; MBI, Maslach Burnout Inventory; WLB, Work–Life Balance; EE, MBI—emotional exhaustion; DEP, MBI—depersonalization; PA, MBI—personal accomplishment.

**Table 2:** Demographic information and test results.

				Female (n = 41)	Male (n = 58)	*p*	Total (n = 99)
Age				37 ± 7	41 ± 8	0.070 ^a^	39.39 ± 7.70
Body Mass Index				25.12 ± 4.42	28.05 ± 4.52	**0.003 ^a^**	26.80 ± 4.68
Title	Research Assistant			9 (22.0%)	10 (17.2%)	0.675 ^b^	19 (41.4%)
	Lecturer			12 (29.3%)	20 (34.5%)		32 (32.3%)
	Asst. Prof			12 (29.3%)	13 (22.4%)		25 (25.3%)
	Assoc. Prof.			5 (12.2%)	6 (10.3%)		11 (11.1%)
	Prof.			3 (7.3%)	9 (15.5%)		12 (12.1%)
Work-related working hours (h/w)				35 ± 14	40 ± 14	0.103 ^a^	37.91 ± 14.14
Computer usage duration (h/w)				31 ± 19	36 ± 23	0.323 ^a^	33.97 ± 21.31
Pain status			No	2 (4.9%)	9 (15.5%)	0.117 ^c^	11 (11.1%)
			Yes	39 (95.1%)	49 (84.5%)		88 (88.9%)
Number of painful regions			0	2 (4.9%)	9 (15.5%)	0.292 ^b^	11 (11.1%)
			1	4 (9.8%)	9 (15.5%)		13 (13.1%)
			2	4 (9.8%)	4 (6.9%)		8 (8.1%)
			3	4 (9.8%)	8 (13.8%)		12 (12.1%)
			≥4	27 (65.9%)	28 (48.3%)		55 (55.6%)
Distribution of regional pain conditions		Neck	No	13 (31.7%)	26 (44.8%)	0.188 ^b^	39 (39.4%)
			Yes	28 (68.3%)	32 (55.2%)		60 (60.6%)
		Shoulder	No	13 (31.7%)	33 (56.9%)	**0.013 ^b^**	46 (46.5%)
			Yes	28 (68.3%)	25 (43.1%)		53 (53.5%)
		Upper Back	No	14 (34.1%)	26 (44.8%)	0.286 ^b^	40 (40.4%)
			Yes	27 (65.9%)	32 (55.2%)		59 (59.6%)
		Elbow	No	34 (82.9%)	43 (74.1%)	0.300 ^b^	77 (77.8%)
			Yes	7 (17.1%)	15 (25.9%)		22 (22.2%)
		Hand	No	21 (51.2%)	36 (62.1%)	0.282 ^b^	57 (57.6%)
			Yes	20 (48.8%)	22 (37.9%)		42 (42.4%)
		Lower Back	No	16 (39.0%)	23 (39.7%)	0.950 ^b^	39 (39.4%)
			Yes	25 (61.0%)	35 (60.3%)		60 (60.6%)
		Hip	No	31 (75.6%)	44 (75.9%)	0.977 ^b^	75 (75.8%)
			Yes	10 (24.4%)	14 (24.1%)		24 (24.2%)
		Knee	No	20 (48.8%)	38 (65.5%)	0.096 ^b^	58 (58.6%)
			Yes	21 (51.2%)	20 (34.5%)		41 (41.4%)
		Feet	No	27 (65.9%)	39 (67.2%)	0.885 ^b^	66 (66.7%)
			Yes	14 (34.1%)	19 (32.8%)		33 (33.3%)
UASWSS				86.02 ± 24.23	73.22 ± 19.00	**0.004 ^a^**	78.42 ± 22.16
MBI—emotional exhaustion				11.07 ± 7.90	9.27 ± 5.92	0.199 ^a^	10.02 ± 6.83
MBI—depersonalization				2.14 ± 2.56	2.31 ± 2.40	0.746 ^a^	2.24 ± 2.45
MBI—personal accomplishment				23.56 ± 4.75	24.05 ± 4.38	0.598 ^a^	23.84 ± 4.52
WLB				39.73 ± 10.92	42.53 ± 10.73	0.207 ^a^	41.57 ± 10.91

h/w: hour/week; UASWSS: the University Academic Staff Work Stress Scale; MBI: The Maslach Burnout Inventory; WLB: The Work–Life Balance Scale; ^a^: Independent samples *t*-test, ^b^: Pearson chi-square test; ^c^: Fisher’s exact test.

**Table 3:** Correlations with the number of painful regions.

	*	Age	BMI	UASWSS	MBI—Emotional Exhaustion	MBI—Depersonalization	MBI—Personal Accomplishment	WLB	Work-Related Working Hours (h/w)	Computer Usage Duration (h/w)
Number of painful regions	*p*	0.227	0.436	**<0.001**	0.249	0.265	**0.037**	**<0.001**	0.085	**<0.001**
	r	−0.129	−0.084	0.663	0.117	0.113	−0.209	−0.596	0.174	0.371

BMI: body mass index; UASWSS: the University Academic Staff Work Stress Scale; MBI: The Maslach Burnout Inventory; WLB: The Work–Life Balance Scale; h/w: hour/week; *: Pearson correlation test.

**Table 4:** Intercorrelations among work-related stress, burnout dimensions, and work–life balance.

	*	UASWSS	MBI—Emotional Exhaustion	MBI—Depersonalization	MBI—Personal Accomplishment	WLB
UASWSS	*p*		**0.001**	**0.016**	0.090	**<0.001**
	r		0.339	0.241	−0.171	−0.412
MBI—emotional exhaustion	*p*			**<0.001**	**0.001**	**0.023**
	r			0.413	−0.332	−0.229
MBI—depersonalization	*p*				0.374	0.778
	r				−0.090	−0.029
MBI—personal accomplishment	*p*					**0.003**
	r					0.293

* Pearson correlation test; UASWSS: the University Academic Staff Work Stress Scale; MBI: The Maslach Burnout Inventory; WLB: The Work–Life Balance Scale.

**Table 5:** Analysis of factors affecting the number of painful regions using ordinal logistic regression.

	B	SE	OR	95% CI (OR)	*p*
Computer usage duration (h/w)	0.041	0.014	1.04	1.01–1.07	**0.003**
UASWSS	0.070	0.017	1.07	1.04–1.11	**<0.001**
MBI—emotional exhaustion	−0.051	0.050	0.95	0.86–1.05	0.311
MBI—depersonalization	−0.086	0.113	0.92	0.73–1.14	0.445
MBI—personal accomplishment	−0.034	0.060	0.97	0.86–1.09	0.568
WLB	−0.100	0.036	0.90	0.84–0.97	**0.006**

h/w: hour/week; UASWSS: the University Academic Staff Work Stress Scale; MBI: The Maslach Burnout Inventory; WLB: The Work-Life Balance Scale; B: regression coefficient; SE: standard error; OR: odds ratio; CI: confidence interval. Model χ^2^ = 71.818, *p* < 0.001; Nagelkerke R^2^ = 0.558. The proportional odds assumption was met (Test of Parallel Lines, *p* = 0.342).

**Table 6:** Comparison of test scores based on the number of painful regions.

	Number of Painful Regions						
	0 (n = 11)	1 (n = 13)	2 (n = 8)	3 (n = 12)	≥4 (n = 55)	*p* ^a^	Eta-Squared (η^2^)
UASWSS	60.54 ± 8.11	63.61 ± 16.73	58.37 ± 18.68	66.75 ± 11.12	90.96 ± 19.46	**<0.001**	0.41
MBI—emotional exhaustion	8.45 ± 5.52	8.15 ± 5.04	7.25 ± 6.75	11.08 ± 4.67	10.94 ± 7.71	0.390	0.04
MBI—depersonalization	2.00 ± 2.52	1.53 ± 2.06	1.75 ± 3.15	3.41 ± 2.87	2.27 ± 2.32	0.379	0.04
MBI—personal accomplishment	26.18 ± 4.11	24.46 ± 4.66	23.62 ± 3.02	25.00 ± 3.86	23.01 ± 4.77	0.214	0.06
WLB	46.72 ± 7.02	49.76 ± 4.14	50.12 ± 3.75	49.58 ± 7.05	41.57 ± 10.91	**<0.001**	0.36

UASWSS: the University Academic Staff Work Stress Scale; MBI: The Maslach Burnout Inventory; WLB: The Work–Life Balance Scale; ^a^: one-way ANOVA test.

## Data Availability

The data presented in this study are available upon reasonable request from the corresponding author. The data are not publicly available due to privacy restrictions.

## Supplementary Materials

The following supporting information can be downloaded at: https://www.mdpi.com/article/10.3390/healthcare14111475/s1, STROBE Statement—checklist of items that should be included in reports of observational studies.
