# Supplementary material for: The Role of Burnout Dimensions, Job Stress, and Work–Life Balance in Multisite Musculoskeletal Pain Among Academicians in Türkiye: A Cross-Sectional Study
Source: Healthcare (Basel). 2026 May 27;14(11):1475. doi: 10.3390/healthcare14111475 (PMC13256750; doi:10.3390/healthcare14111475)
Supplement: Supplementary file 1 [file healthcare-14-01475-s001.zip › healthcare-4192595-supplementary.pdf]

STROBE Statement—checklist of items that should be included in reports of observational studies

	Item No.	Recommendation	Page No.	Relevant text from manuscript
**Title and abstract**	1	(*a*) Indicate the study's design with a commonly used term in the title or the abstract	1	The design "Cross-Sectional Study" is explicitly stated in both the title and the abstract.
		(*b*) Provide in the abstract an informative and balanced summary of what was done and what was found	1	abstract
**Introduction**				
Background/rationale	2	Explain the scientific background and rationale for the investigation being reported	2-4	All introduction
Objectives	3	State specific objectives, including any prespecified hypotheses	4	Therefore, our study aims to investigate the relationship between MSDs and academic stress, burnout, and work-life balance among academicians.
**Methods**				
Study design	4	Present key elements of study design early in the paper	4	The Methods section states that this was a cross-sectional study conducted between January and July 2025 at Yalova University.
Setting	5	Describe the setting, locations, and relevant dates, including periods of recruitment, exposure, follow-up, and data collection	4	The study was conducted among academicians working at Yalova University between January 2025 and July 2025
Participants	6	(*a*) *Cohort study*—Give the eligibility criteria, and the sources and methods of selection of participants. Describe methods of follow-up<br>*Case-control study*—Give the eligibility criteria, and the sources and methods of case ascertainment and control selection. Give the rationale for the choice of cases and controls<br>*Cross-sectional study*—Give the eligibility criteria, and the sources and methods of selection of participants	5	The inclusion criteria for the study were: volunteering to participate, having worked as academic staff at Yalova University for the past six months, and being able to

				understand and speak Turkish language. Individuals who wished to withdraw from the study, those with a history of fracture or soft tissue injury in any body region within the past 12 months, congenital spinal disorders, scoliosis, rheumatoid diseases, cancer, surgery, chronic internal organ pain, long-term use of analgesic or psychiatric medications, and those working in departments without students were excluded from the study.
		(*b*) *Cohort study*—For matched studies, give matching criteria and number of exposed and unexposed<br>*Case-control study*—For matched studies, give matching criteria and the number of controls per case		
Variables	7	Clearly define all outcomes, exposures, predictors, potential confounders, and effect modifiers. Give diagnostic criteria, if applicable	5-6	Outcomes: number of painful regions from NMQ-E. Predictors: job stress (UASWSS), burnout (MBI), work-life balance (WLB), and computer usage.
Data sources/ measurement	8*	For each variable of interest, give sources of data and details of methods of assessment (measurement). Describe comparability of assessment methods if there is more than one group	5-6	Standardized, validated Turkish versions of NMQ-E, UASWSS, MBI, and WLB scales were used. Measurements are self-reported

			questionnaires.
Bias	9	Describe any efforts to address potential sources of bias	6 All participants were included based on predefined inclusion and exclusion criteria, and validated measurement tools were used. Data collection and analyses were performed by the research team to ensure objectivity. Therefore, no potential sources of bias were identified in this study.
Study size	10	Explain how the study size was arrived at	5 The study population consisted of all academicians working at Yalova University. The sample included those who voluntarily agreed to participate, and therefore no specific sample size calculation was performed.

Continued on next page

Quantitative variables	11	Explain how quantitative variables were handled in the analyses. If applicable, describe which groupings were chosen and why	6	Quantitative variables (age, BMI, test scores) were expressed as mean ± SD. Groupings were based on sex and the number of painful regions (0, 1, 2, 3, 3+), representing MSD severity levels.
Statistical methods	12	(*a*) Describe all statistical methods, including those used to control for confounding	6	SPSS 26.0 used. Categorical data: Chi-Square/Fisher tests. Continuous data: Independent t-test. Correlations: Pearson. Group comparisons: One-Way ANOVA + Tamhane. Ordinal regression used for predictors. $p < 0.05$ considered significant.
		(*b*) Describe any methods used to examine subgroups and interactions	6	
		(*c*) Explain how missing data were addressed	6	No data were lost during the study. All participants completed the assessments, and the dataset was analyzed in full.
		(*d*) *Cohort study*—If applicable, explain how loss to follow-up was addressed<br>*Case-control study*—If applicable, explain how matching of cases and controls was addressed<br>*Cross-sectional study*—If applicable, describe analytical methods taking account of sampling strategy	6	The study included all academicians working at Yalova University as the target population, and the sample consisted of volunteers who met the inclusion criteria. Since no complex or stratified sampling strategy was applied, no weighting or adjustment for sampling design was required in the analyses.
		(*e*) Describe any sensitivity analyses		
**Results**				

Participants	13*	(a) Report numbers of individuals at each stage of study—eg numbers potentially eligible, examined for eligibility, confirmed eligible, included in the study, completing follow-up, and analysed	7	Of 620 eligible academicians, 521 were excluded per criteria or declined participation. 99 participants were included; no missing data.
		(b) Give reasons for non-participation at each stage	7	Of 620 academicians reached, 521 were excluded for not meeting the inclusion criteria (those with musculoskeletal or psychiatric conditions, those in departments without actively enrolled students, those with less than six months of work experience, and individuals who declined to participate)."
		(c) Consider use of a flow diagram		
Descriptive data	14*	(a) Give characteristics of study participants (eg demographic, clinical, social) and information on exposures and potential confounders	7-8	Table 1
		(b) Indicate number of participants with missing data for each variable of interest	7	No data were lost during the study. All participants completed the assessments, and the dataset was analyzed in full.
		(c) *Cohort study*—Summarise follow-up time (eg, average and total amount)		
Outcome data	15*	*Cohort study*—Report numbers of outcome events or summary measures over time		
		Case-control study—Report numbers in each exposure category, or summary measures of exposure		
		Cross-sectional study—Report numbers of outcome events or summary measures	6-7	
Main results	16	(*a*) Give unadjusted estimates and, if applicable, confounder-adjusted estimates and their precision (eg, 95% confidence interval). Make clear which confounders were adjusted for and why they were included	8-9	Correlation and regression analyses (Tables 2–3) report p-values and confidence intervals for predictors of MSDs.
		(*b*) Report category boundaries when continuous variables were categorized		
		(*c*) If relevant, consider translating estimates of relative risk into absolute risk for a meaningful time		

		period		
Other analyses	17	Report other analyses done—eg analyses of subgroups and interactions, and sensitivity analyses	9	Subgroup comparisons made by sex and by number of painful regions (Table 4).
**Discussion**				
Key results	18	Summarise key results with reference to study objectives	10	High MSD prevalence (88.9%), job stress, and impaired work-life balance are strongly related to MSD severity.
Limitations	19	Discuss limitations of the study, taking into account sources of potential bias or imprecision. Discuss both direction and magnitude of any potential bias	14	
Interpretation	20	Give a cautious overall interpretation of results considering objectives, limitations, multiplicity of analyses, results from similar studies, and other relevant evidence	10/14	Results interpreted within the dual physical–psychosocial pathway framework; findings compared to national and international literature.
Generalisability	21	Discuss the generalisability (external validity) of the study results	12/13	Results are specific to Yalova University academicians but may reflect psychosocial risks in similar academic environments.
**Other information**				
Funding	22	Give the source of funding and the role of the funders for the present study and, if applicable, for the original study on which the present article is based	15	"This research received no specific grant from any funding agency." stated under *Declarations.*

*Give information separately for cases and controls in case-control studies and, if applicable, for exposed and unexposed groups in cohort and cross-sectional studies.

Note: An Explanation and Elaboration article discusses each checklist item and gives methodological background and published examples of transparent reporting. The STROBE checklist is best used in conjunction with this article (freely available on the Web sites of PLoS Medicine at http://www.plosmedicine.org/, Annals of Internal Medicine at http://www.annals.org/, and Epidemiology at http://www.epidem.com/). Information on the STROBE Initiative is available at www.strobe-statement.org.
